# Comparative evaluation and performance of large language models on expert level critical care questions: a benchmark study

**DOI:** 10.1186/s13054-025-05302-0

**Published:** 2025-02-10

**Authors:** Jessica D. Workum, Bas W. S. Volkers, Davy van de Sande, Sumesh Arora, Marco Goeijenbier, Diederik Gommers, Michel E. van Genderen

**Affiliations:** 1 Department of Adult Intensive Care, Erasmus MC University Medical Center, Rotterdam, The Netherlands; 2 Department of Intensive Care, Elisabeth-TweeSteden Hospital, Tilburg, The Netherlands; 3 Erasmus MC Datahub, Erasmus MC University Medical Center, Rotterdam, The Netherlands; 4 Prince of Wales Hospital, Sydney, Australia; 5 Department of Intensive Care Medicine, Spaarne Gasthuis, Haarlem, Hoofddorp, The Netherlands

**Keywords:** Large language models, Generative artificial intelligence, Critical care, Benchmarking

## Abstract

**Background:**

Large language models (LLMs) show increasing potential for their use in healthcare for administrative support and clinical decision making. However, reports on their performance in critical care medicine is lacking.

**Methods:**

This study evaluated five LLMs (GPT-4o, GPT-4o-mini, GPT-3.5-turbo, Mistral Large 2407 and Llama 3.1 70B) on 1181 multiple choice questions (MCQs) from the gotheextramile.com database, a comprehensive database of critical care questions at European Diploma in Intensive Care examination level. Their performance was compared to random guessing and 350 human physicians on a 77-MCQ practice test. Metrics included accuracy, consistency, and domain-specific performance. Costs, as a proxy for energy consumption, were also analyzed.

**Results:**

GPT-4o achieved the highest accuracy at 93.3%, followed by Llama 3.1 70B (87.5%), Mistral Large 2407 (87.9%), GPT-4o-mini (83.0%), and GPT-3.5-turbo (72.7%). Random guessing yielded 41.5% (*p* < 0.001). On the practice test, all models surpassed human physicians, scoring 89.0%, 80.9%, 84.4%, 80.3%, and 66.5%, respectively, compared to 42.7% for random guessing (*p* < 0.001) and 61.9% for the human physicians. However, in contrast to the other evaluated LLMs (*p* < 0.001), GPT-3.5-turbo’s performance did not significantly outperform physicians (*p* = 0.196). Despite high overall consistency, all models gave consistently incorrect answers. The most expensive model was GPT-4o, costing over 25 times more than the least expensive model, GPT-4o-mini.

**Conclusions:**

LLMs exhibit exceptional accuracy and consistency, with four outperforming human physicians on a European-level practice exam. GPT-4o led in performance but raised concerns about energy consumption. Despite their potential in critical care, all models produced consistently incorrect answers, highlighting the need for more thorough and ongoing evaluations to guide responsible implementation in clinical settings.

**Supplementary Information:**

The online version contains supplementary material available at 10.1186/s13054-025-05302-0.

## Introduction

Large Language Models (LLMs), a subclass of artificial intelligence (AI) systems that can generate human-like natural language responses to text, hold great promise in supporting healthcare professionals with administrative tasks, such as summarizing clinical notes and drafting answers to patient questions [1]. Additionally, when aiding healthcare professionals with informed medical decisions, we recognize them as AI—clinical decisions support (AI-CDS) tools [2, 3]. Given the complexity and high stakes of such decision-making, ensuring the accuracy and reliability of these systems is paramount, particularly within domains that require highly specific expertise [4].

Clinical decision-making is a multifaceted process that requires medical knowledge, clinical reasoning, the capacity to integrate and synthesize information from various disciplines, and the ability to apply evidence-based practice. However, as a first step for LLMs towards being effective in clinical decision-making, possessing inherent medical knowledge is a fundamental requirement. In recent studies, the medical performance of non-domain specific LLMs was evaluated and findings showed that they can pass the United States Medical Licensing Examination (USMLE) [5, 6]. However, to move towards utilizing LLMs for clinical decision-making in highly specific healthcare fields, where clinical decisions are strongly dependent on a continuous influx of new clinical trial data, it is critical to benchmark LLM models and describe their performance characteristics to guide their safe application in more specific and conceptually complex medical areas. Recent studies evaluated these models in the fields of nephrology and oncology, benchmarking several LLMs using multiple choice questions (MCQs) in their respective field, in which the GPT-4 model typically outperformed the other models [7–9]. Similar studies in other specialties have shown comparable trends, with newer models generally performing superiorly [10, 11].

In the critical care medicine, unique challenges present themselves that require rapid decision-making, comprehensive knowledge, and the ability to integrate complex information from multiple organ systems [12]. Currently, a structured assessment of the performance of LLMs in critical care medicine is lacking.

The aim of this study was therefore to evaluate and compare the performance of various LLMs on answering high level critical care questions and compare their performance to human physicians. We also determined costs as a proxy for energy consumption. Our goal was to understand the accuracy, consistency, and limitations of LLMs applied to critical care medicine to guide future studies and applications.

## Methods

We performed this cross-sectional comparative study in October 2024. The study was exempt from research ethics board approval and the need for informed consent in accordance with European law, given the lack of involvement of human participants or patient data. We utilized the TRIPOD-LLM guideline for reporting [13].

### Dataset

This study utilized 1181 critical care MCQs from the gotheextramile.com (GETM) dataset [14]. GTEM is a comprehensive dataset, designed to assess critical care knowledge at the European Diploma in Intensive Care (EDIC) examination level. Similar to the EDIC exam, the dataset included two types of questions: 74.5% (n = 880) were Type A MCQs (single best answer from four options) and 25.5% (n = 301) were Type K MCQs (four or five true or false statements). The GTEM dataset covers a wide range of critical care domains, which can be found in Supplement A. Due to the nature of the specific LLMs tested, image-based questions were omitted.

### Human comparison

For the comparison with human physicians, we evaluated the LLMs on a practice exam at EDIC examination level, consisting of 77 questions, of which 68.8% (n = 53) were Type A and 31.2% (n = 24) were Type K MCQs. For this exam, performance was calculated using a scoring system, in which false answers would deduct points. Human comparators were GTEM subscribers who took the practice exam in 2023 or 2024.

GTEM’s subscriber base spans roughly 50 countries, with India accounting for the largest portion (40–50%). European nations, notably the Netherlands, Denmark, Switzerland, Germany, and the UK, make up the second-largest group, while the United States has minimal representation. The subscriber base consists of physicians, both residents and consultants, predominantly those preparing for the written EDIC exam. Subscribers typically have backgrounds in anesthesiology, with 3 to 5 years of experience in anesthesiology and 1 to 5 years in intensive care. Approximately 30% are repeat subscribers.

### Large language models

In total, five LLMs were evaluated: four proprietary foundation models, i.e. GPT-4o, GPT-4o-mini and GPT-3.5-turbo, developed by OpenAI, and Mistral Large 2407 (Mistral AI), and one open-source model, being Llama 3.1 70B (Meta). They were accessed via the Microsoft Azure OpenAI platform. For each model, the temperature setting, which controls the randomness of the model’s outputs, was set to 0, to achieve the most consistent results.

### Prompting methods

The prompts were written by a team of experts in medical prompt engineering in an iterative manner. The final prompt used in this study is available in Supplement B. It was important for the final prompt to have utilized zero-shot prompting, meaning that the models are presented with critical care MCQs without prior fine-tuning on medical datasets or knowledge of example questions. This approach aims to assess the models’ inherent critical care knowledge and provide accurate answers based solely on their pre-trained knowledge.

### Evaluation

The performance of the LLMs was assessed using multiple evaluation metrics. The primary outcome measure was the overall accuracy, calculated as the percentage of correctly answered MCQs, which we compared to random guessing (e.g., 25% accuracy for questions with four options).

Secondary outcomes included consistency, domain-specific accuracy, costs as a proxy for computing resources or energy use and performance of the LLMs compared with human physicians on a practice exam.

A consistency check was performed by presenting the models with repeated questions to assess the reliability of model responses. One hundred randomly selected questions from the dataset were given to each model 10 times. A model’s response was considered consistent if it provided the same answer for at least 8 out of 10 repetitions (80% threshold) of a given question. The consistency score for each model was calculated as the percentage of questions for which the model demonstrated consistency, distinguishing between consistently correct and consistently incorrect responses.

Domain-specific performance was evaluated by categorizing questions according to critical care topics and calculating accuracy rates for each domain.

Cost per model were calculated directly using the Microsoft Azure OpenAI platform. Pricing is based on the amount of input and output tokens. Efficiency scores were calculated by dividing the performance of the model with its cost.

### Statistical analyses

All statistical analyses were performed with Python version 3.11, utilizing libraries such as Numpy, Pandas, Matplotlib, Seaborn and Statsmodels. Continuous variables were summarized using means and standard deviations (SD) or medians and interquartile ranges (IQR), based on the distribution of the data. Categorical variables were summarized as percentages.

The primary outcome measure, overall accuracy, was calculated as the percentage of correctly answered MCQs for each model. Comparative analyses between the performance of the LLMs against random guessing and human physician comparators were conducted using Z-tests for proportions. This method was selected due to the large sample size and binary nature of outcomes (correct/incorrect), allowing for reliable comparisons against fixed performance benchmarks (i.e. random guessing or human comparators).

A *p* value of < 0.05 was considered statistically significant.

## Results

Our results show that the GPT-4o model was the most accurate, correctly answering 93.3% of the questions. For the other models, GPT-4o-mini answered 83.0%, GPT-3.5-turbo 72.7%, Llama 3.1 70B 87.5%, and Mistral Large 2407 87.9% of the answers correctly (Table 1). Considering the number of questions in the dataset and the choices per question, we calculated a performance of 41.5% by random guessing. When compared to random guessing, all models show statistically significant superior performance (*p* < 0.001).

**Table 1:** Performance (percentage answered correctly), cost (as a proxy for energy use) and efficiency scores for the various LLMs on all MCQs. Efficiency is the ratio of performance (accuracy) to cost, reflecting the balance between performance and resource usage

Model	Performance (%)	Cost	Efficiency score
GPT-4o	93.3	€ 3.60	25.9
GPT-4o-mini	83.0	€ 0.14	592.9
GPT-3.5-turbo	72.7	€ 0.96	75.7
Llama 3.1 70B	87.5	€ 1.80	48.6
Mistral Large 2407	87.9	€ 2.70	32.6

All models demonstrated high consistency in their performance (Table 2). Mistral Large 2407 and GPT-4o showed the highest consistency, scoring 100% and 96.0% respectively, providing identical answers in at least 8 out of 10 repetitions for nearly all 100 questions. GPT-4o-mini and Llama 3.1 70B followed closely, scoring 93.0% and 92.0%, while GPT-3.5-turbo showed the lowest consistency at 74.0%. We assessed if the consistent answers were correct or incorrect, relating accuracy and consistency. A high consistently correct score shows that a model is not only accurate but also reliably produces the correct answer when asked multiple times. GPT-4o showed the highest consistently correct score of 88.5% and GPT-3.5-turbo had the least score of 67.6% (Table 2).

**Table 2:** Consistency scores for all models. Consistency reflects the percentage (%) of questions for which a model provided the same answer in at least 8 out of 10 repetitions, from a random subset of 100 questions. Consistently correct and consistently incorrect scores indicate the proportion of these responses that were accurate or erroneous, respectively

Model	Consistency (%)	Consistenly correct (%)	Consistenly incorrect (%)
GPT-4o	96.0	88.5	11.5
GPT-4o-mini	93.0	76.3	23.7
GPT-3.5-turbo	74.0	67.6	32.4
Llama 3.1 70B	92.0	81.5	18.5
Mistral Large 2407	100.0	81.0	19.0

**Fig. 1:**
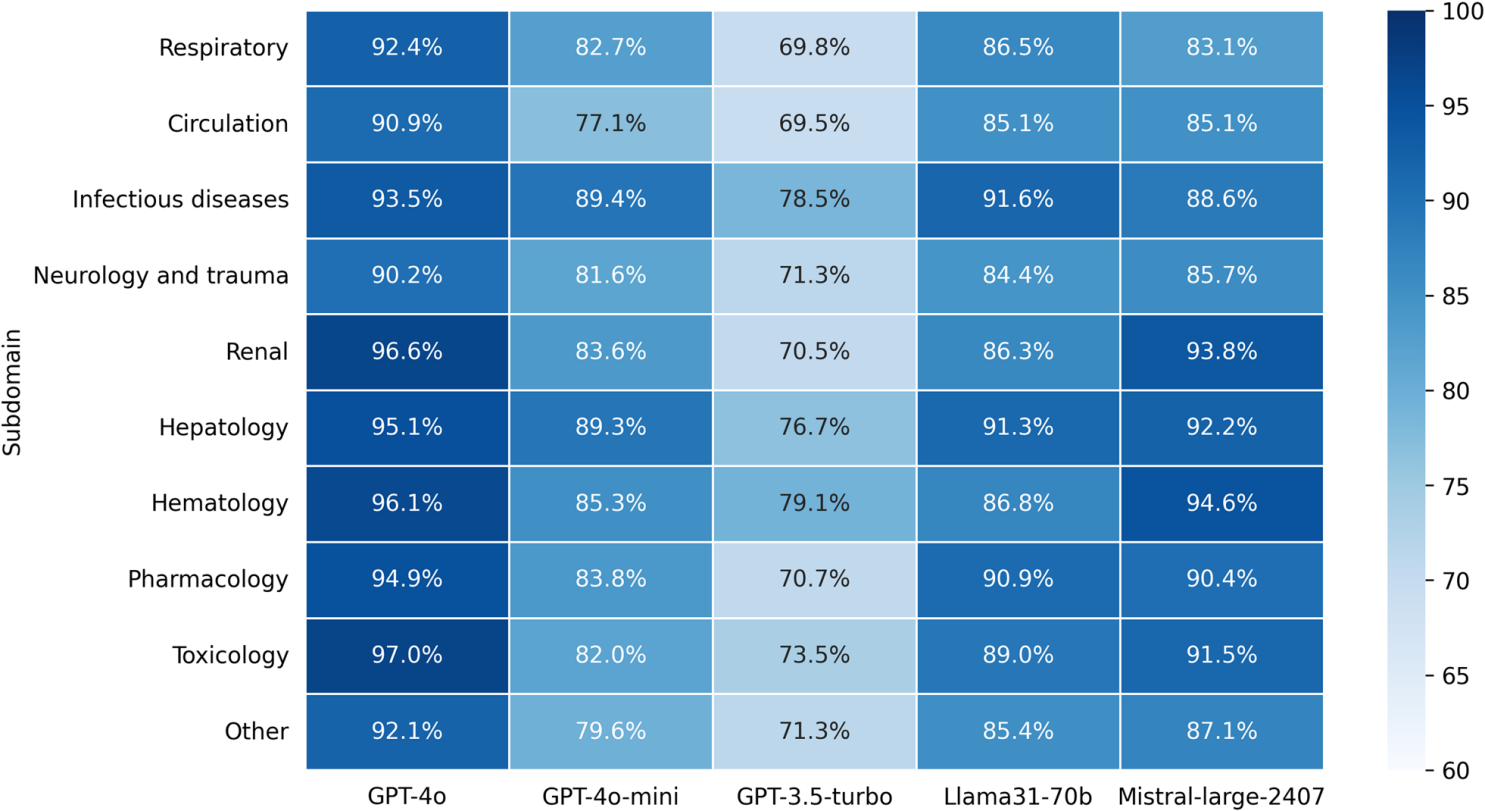
Performance per subdomain

Furthermore, we evaluated the performance for each model for the various critical care subdomains (Fig. 1). For each subdomain, the GPT-4o model scored the highest. Over the various subdomains, all models showed low variability, with a standard deviation of 2.3 for GPT-4o, 3.7 for GPT-4o-mini, 3.5 for GPT-3.5-turbo, 2.6 for Llama 3.1 70B, and 3.7 for Mistral Large 2407. This indicates consistent subdomain knowledge in all tested models. The lowest scoring domains differed between the various tested models, indicating no specific gap in medical subdomain knowledge across LLMs.

**Fig. 2:**
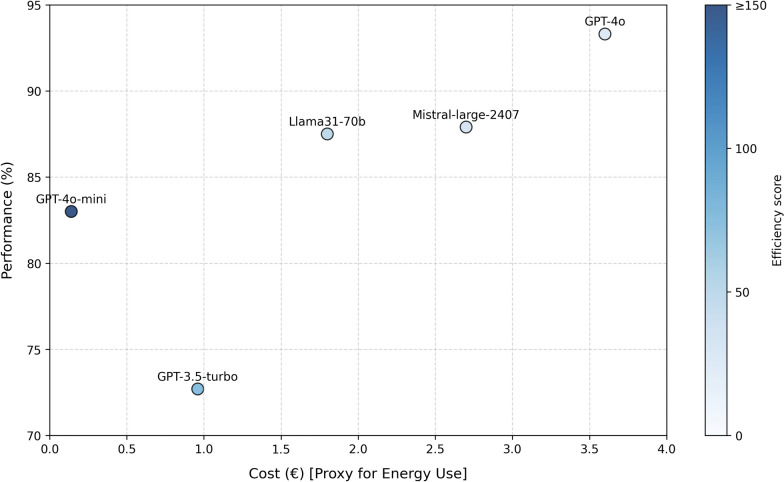
Comparing performance and cost (as a proxy for energy use) of for the different LLMs

As a proxy for computing resources, i.e. energy use, token cost was calculated per model for all 1181 questions. The GPT-4o-mini model was the cheapest, costing €0.14 to run, whereas the GPT-4o model was the most expensive (€3.60). GPT-4o-mini achieves the strongest balance between energy use and performance, offering close-to-top-tier results while requiring much less computational power, reflected in its low cost (Fig. 2). All costs and efficiency scores can be found in Table 1.

**Fig. 3:**
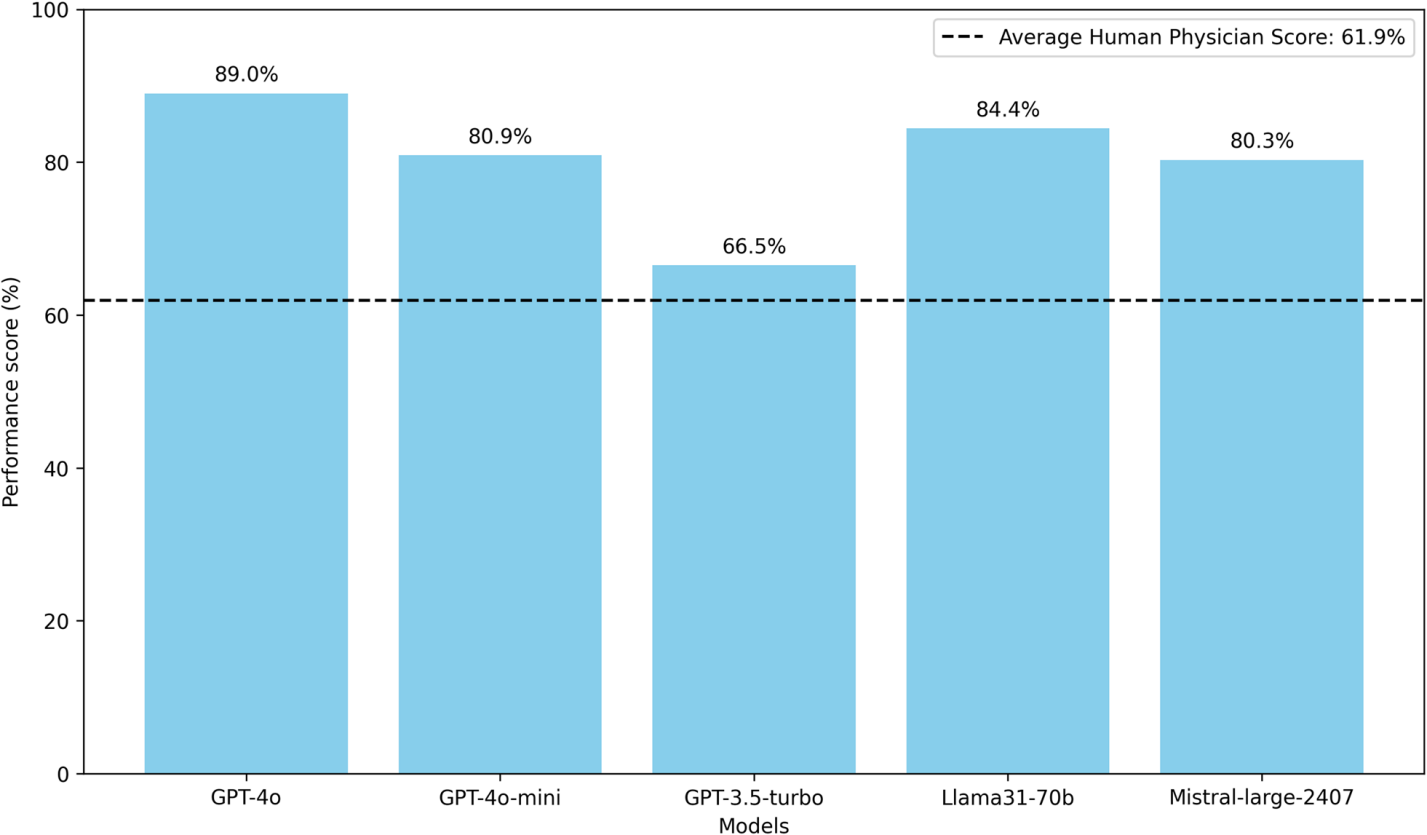
Performance score of LLMs versus human physicians on practice exam

In the EDIC-like practice exam comparison with human performance, GPT-4o had the highest performance score of 89.0% and GPT-3.5-turbo had the lowest with 66.5% (Fig. 3). All LLMs demonstrated statistically significant superior performance compared to random guessing, calculated as 42.7% (*p* < 0.001). Between January 2023 and October 2024, 350 physicians completed the practice exam, with an average score of 61.9%. All LLMs outperformed the human physician comparators. However, in contrast to the other evaluated LLMs (*p* < 0.001), the performance of GPT-3.5-turbo compared to human physicians did not reach statistical significance (*p* = 0.196).

## Discussion

In this study, we evaluated five LLMs, both proprietary and open source, on a large dataset of critical care questions at the European examination level. Our main findings show that all tested LLMs have a high accuracy and as such an outstanding performance. The GPT-4o model had the best performance with 93.3% of questions answered correctly, and significantly outperforms the human physicians. This indicates that critical care domain knowledge is inherently present in these LLMs, potentially rendering them suitable for prospective clinical trials focused on utilizing LLMs in critical care for clinical decision-making.

When compared to the benchmarking studies performed in other highly specific fields, such as nephrology and oncology [7, 8], our results show significantly higher performance of the different models. It is important to note that the best performing model (GPT-4o) is a different model than the best performing models used in those studies (GPT-4). GPT-4o has been shown to have higher performance than GPT-4, which has also been demonstrated in the medical field. For instance, Liu et al. evaluated the performance of various models, including GPT-4o and GPT-4, on the Japanese Medical Licensing Examination and found that GPT-4o outperformed all other tested models with an accuracy of 89.2%, whereas GPT-4 had a significantly lower performance with 76.8% correct [15].

We also evaluated the consistency of the various models and found very high consistency in at least 2 models, i.e. Mistral Large 2407 and GPT-4o. However, for clinical practice, consistency alone is not sufficient to determine the reliability of a model, as it could be consistently incorrect. Consistently incorrect scores were observed in all models. Notably, despite a consistency of 100%, Mistral Large 2407 was consistently incorrect in 19.0% of cases. The GPT-3.5-turbo model showed both the lowest consistency (74.0%) and was only consistently correct in 67.6% of cases. An LLM used in clinical practice should show both high accuracy and high consistency. However, consistently incorrect scores answers raise critical safety concerns particularly relevant to critical care, where dynamic evidence informs decisions with high-stakes consequences. Future research should evaluate techniques to improve safety and should take consistency into account when performing prospective clinical trials with LLMs.

Additionally, we calculated token cost for all models as a proxy for computing resources or energy consumption and as such sustainability. In LLMs, tokens represent a small piece of text to process the input and generate a response, by predicting one token at a time, sequentially. The computational requirements and, consequently, energy consumption is directly proportional to the number of tokens utilized by a model [16, 17]. Furthermore, the size of the LLM is also a significant contributor to energy consumption, with larger models generally consuming more energy per token than their smaller counterparts [18]. LLM token costs could serve as surrogate for energy consumption and as such an indirect measure of sustainability. The most expensive model was GPT-4o, costing over 25 times more than the least expensive model, GPT-4o-mini. However, GPT-4o-mini was 10.3% less accurate than GPT-4o in answering the MCQs correctly. When balancing costs, and thus energy consumption, with performance, GPT-4o-mini yields the highest efficiency. For the implementation of LLMs in healthcare settings, it is crucial to carefully weigh the trade-offs between energy consumption and performance capabilities, including accuracy and consistency. For use cases where inherent medical domain knowledge might be of less importance, such as using LLMs for administrative support, opting for a more cost-effective model might be a more judicious approach. However, even when not used for clinical decision making, assessing accuracy, consistency and clinical validation of the LLM for each use case remain essential steps for the safe implementation of LLMs in healthcare.

While this the first study in its kind to benchmark various LLMs in critical care against examination-level MCQs, several limitations should be acknowledged. First, our study focused on MCQs and omitted image-based questions, which does not fully capture the complexity and depth of understanding required in real-world clinical decision-making. While MCQs are a useful tool for assessing basic knowledge, we did not evaluate the reasoning process behind its decision-making. As a result, the models’ ability to handle more intricate or open-ended problem-solving scenarios, where clinical reasoning is critical, remains untested. Studies on the clinical reasoning capabilities of LLMs have reported promising results, with the GPT-4 model scoring between 66.7 and 76.0% on various general medical questions and clinical cases [19, 20]. However, they are prone to overconfidence and exhibit cognitive biases [21, 22]. This furthermore underscores the importance of thorough and ongoing clinical validation before use these models as clinical decision support tools. For highly specialized subdomains like critical care, these studies are lacking. However, we are currently conducting an evaluation study on the reasoning capabilities of LLMs in critical care medicine to further explore their potential in clinical decision making.

Second, there is a risk of potential data leakage, meaning that questions from the GTEM dataset may have been inadvertently present in the training set of the models. If any of the test questions or their near equivalents were present in the models’ training data, this could artificially inflate their performance. However, given that GTEM is a protected dataset, this is not considered likely. Last, human comparators were physicians preparing for their exam and may have less critical care knowledge and experience than seasoned critical care specialists. However, participating physicians had 1–5 years of experience in critical care.

## Conclusion

Our study showed that all tested LLMs have a high accuracy and consistency in answering critical care medicine questions at European examination level. Additionally, four out of five LLMs demonstrated superior performance compared to human physicians on a practice exam, indicating a substantial potential for utilizing these models in critical care. Notably, the GPT-4o model outperformed all other models in terms of accuracy and consistency, yet it was also the most expensive model, suggesting higher energy consumption. When utilizing LLMs in healthcare environments, it is essential to balance energy usage against performance metrics and selecting a more economical model may be the more prudent choice. Despite their potential in critical care, all models produced consistently incorrect answers, highlighting the need for more thorough and ongoing evaluations, with a focus on clinical reasoning capabilities, to guide responsible implementation in clinical settings.

## Supplementary Information

Supplementary Material file 1.

## Data Availability

The questions of gotheextramile.com remain confidential.

## Code availability

The prompt is attached in the supplementals. Additional information is available upon request.
