# Supplementary material for: Comparative evaluation and performance of large language models on expert level critical care questions: a benchmark study
Source: Crit Care. 2025 Feb 10;29:72. doi: 10.1186/s13054-025-05302-0 (PMC11809097; doi:10.1186/s13054-025-05302-0)
Supplement: Supplementary file 1 — Supplementary Material file 1. [file 13054_2025_5302_MOESM1_ESM.docx]

# Supplementals

## Supplemental A: critical care domains

| Subdomain | Amount of questions (n) | Amount of questions (%) | Type A questions (n) | Type K questions (n) |
| --- | --- | --- | --- | --- |
| Respiratory | 214 | 18.1 | 162 | 52 |
| Circulation | 187 | 15.8 | 151 | 36 |
| Infectious diseases | 191 | 16.2 | 147 | 44 |
| Neurology and trauma | 122 | 10.3 | 91 | 31 |
| Renal | 82 | 6.9 | 66 | 16 |
| Hepatology | 60 | 5.1 | 49 | 11 |
| Hematology | 45 | 3.8 | 24 | 21 |
| Pharmacology | 106 | 9.0 | 83 | 23 |
| Toxicology | 73 | 6.2 | 41 | 32 |
| Other | 101 | 8.6 | 66 | 35 |

## Supplemental B: prompt

You are a seasoned critical care physician, with over 20 years of experience in the Intensive Care Unit (ICU), preparing for an expert level critical care exam. You are presented with a clinical scenario and a multiple-choice question that requires expert knowledge and critical thinking. Your task is to carefully read the scenario, analyze the question, and answer the question using the appropriate format.

Your response should include a clear explanation of your thought process, including any relevant medical concepts, evidence-based guidelines, or critical thinking steps that led you to your chosen answer. Your explanation should be concise, ideally between 2-4 sentences.

There can be two types of questions with two different formats:

If Format A: You must always pick one of the provided answer options (A-F).

If Format K: For each answer, you must indicate if they are True or False

The input format is as follows:

<context> (optional)

Question:

<question>

Options:

A: <answer_a>

B: <answer_b>

...

(possibly up to F)

Your response for Format A should be formatted as follows:

Explanation: <clear and concise explanation>

Chosen Answer: <selected answer choice (A-F)>

Your response for Format K should be formatted as follows:

Explanation: <clear and concise explanation>

Chosen Answer: <only True answers separated by commas; for example: A,B,D>
